# Triple-mode squeezing with dressed six-wave mixing

**DOI:** 10.1038/srep25554

**Published:** 2016-05-12

**Authors:** Feng Wen, Zepei Li, Yiqi Zhang, Hong Gao, Junling Che, Junling Che, Hasan Abdulkhaleq, Yanpeng Zhang, Hongxing Wang

**Affiliations:** 1 Key Laboratory for Physical Electronics and Devices of the Ministry of Education & Shaanxi Key Lab of Information Photonic Technique, Xi’an Jiaotong University, Xi’an 710049, China; 2 School of Science, Xi’an Jiaotong University, Xi’an 710049, China

## Abstract

The theory of proof-of-principle triple-mode squeezing is proposed via spontaneous parametric six-wave mixing process in an atomic-cavity coupled system. Special attention is focused on the role of dressed state and nonlinear gain on triple-mode squeezing process. Using the dressed state theory, we find that optical squeezing and Autler-Towns splitting of cavity mode can be realized with nonlinear gain, while the efficiency and the location of maximum squeezing point can be effectively shaped by dressed state in atomic ensemble. Our proposal can find applications in multi-channel communication and multi-channel quantum imaging.

Generating squeezed vacuum and entanglement with controllable quantum states is important to quantum communication, quantum information processing and quantum computation^1^,^2^,^3^. For example, the single-mode quadrature squeezed state is used for gravity wave detection, which is produced by the highly refined optical parametric oscillators (OPOs)^4^. The linear optical processing of single-beam quadrature squeezed states is used for the continuous-variable quantum computing^5^,^6^. The multi-spatial mode squeezed light is desirable to continuous-variable quantum image processing techniques^7^,^8^. Generally speaking, techniques for producing the squeezing states are based on either parametric down conversion in solid state crystal or spontaneous parametric four-wave mixing (SP-FWM) schemes in atomic vapors. Traditionally, biphotons generated from Spontaneous parametric down-conversion (SPDC) in nonlinear crystals have very wide bandwidth (THz) and ultra-short coherence time (ps)^9^,^10^.

Using four-wave mixing (FWM) in sodium vapor, squeezed state of light was experimentally implemented^11^,^12^,^13^, however, achieving higher degree of squeezing via FWM in atomic vapors is limited by spontaneous emission noise. Recently, researches on FWM in atomic vapors demonstrated that the spontaneous emission noise can be reduced or eliminated by using of electromagnetically induced transparency (EIT)^14^. In EIT window the transmission^1^, slowing down^15^,^16^,^17^, and storage and retrieval^18^,^19^ of squeezed states were also experimentally demonstrated. Nowadays, the interest to generate triple-mode squeezing is mainly due to its applicability in quantum information and communication^20^,^21^ where the field fluctuations in one of the quadratures are reduced below the vacuum noise level, and can be used in overcoming the shot-noise precision restrictions in optical measurements^22^ and enhancing the capacity of communication channels^23^. Usually, signal-to-noise ratio is very low for six-wave mixing (SWM) in atomic vapor. However, by employing two-photon Doppler-free configurations as well as EIT, enhanced nonlinear processes due to atomic coherence have been experimentally demonstrated^24^. The essentials of such enhanced nonlinear optical processes are the enhanced nonlinear susceptibility due to atomic coherence, slowed light beam propagation in the atomic medium, and greatly reduced linear absorption of the generated optical field due to EIT, which enable us to investigate the topological photonic problems in atomic ensembles^25^,^26^.

In our previous studies, distinctly different from and advantageous over the previously reported^27^, highly efficient FWM, SWM, and eight-wave mixing processes was experimentally demonstrated in an open-cycle Y-type atomic system. By manipulating the atomic coherence and multi-photon interferences among different energy levels, we also demonstrated that the third-order and fifth-order nonlinear processes can coexist in open (such as V-type, Y-type, and inverted Y-type) atomic systems^24^, and the SWM signal can be comparable with or even greater than the FWM signal in amplitude. Such coexisting processes allow us to investigate spatial-temporal coherent interference between third-order and fifth-order nonlinear processes. We also demonstrated the parametrically amplified FWM (PA-FWM) and parametrically amplified SWM (PA-SWM) processes. Such enhanced nonlinear process^28^ is used to generate the strongly correlated bright twin or triple-mode bright beams in cavity and free space with high efficiency and narrow band width.

In this paper, by applying dressed state theory, we examine the influence of dressed state and nonlinear gain on the triple-mode squeezing via three-mode cone emission of SP-SWM, and single-mode and two-mode squeezing via degenerate and nondegenerate self-diffraction (or phase-conjugate) cone emissions of SP-FWM. It is indicated that the optical squeezing and nonlinear Autler-Towns (AT) splitting of quantum noise can be achieved via nonlinear gain. The profiles and location of maximum squeezing point can be effectively modulated by dressed state, that can be achieved only in atomic media. The triple-mode squeezing state proposed in current work can be directly used in multichannel quantum imaging. The quality of imaging (e.g. the contrast and resolution) is significantly improved, compared with that obtained by using the two-mode squeezing state. In addition, the work can be used as implementation of triple-mode entangled source, where the generation efficiency of entangled triple-beam and degree of entanglement will be significantly enhanced by nonlinear susceptibility and quantum gain. Therefore, our scheme can be also used to achieve multi-channel communication. Finally, by using SP-SWM both in cold and hot atomic ensembles, narrow-band triple-photons with a long coherence time is realized. Such a long coherence time may allow us to access and manipulate the squeezed state directly, and has potential application in the long-distance quantum communication.

So our scheme has following advantages. First, due to the near- and on-resonance nonlinear optical processes can be enhanced by atomic coherence technique, so the generation efficiency of SP-SWM and degree of triple of squeezing (entanglement) can be significantly enhanced by manipulating nonlinear susceptibility and quantum gain. Second, due to EIT window (MHz), not only the resonance absorption is eliminated, but narrow bandwidth signals at low light level is obtained as well. So compared with correlated photon pairs from SPDC, which have characteristics of very wide bandwidth (THz) and ultra-short coherence time (ps). Our scheme has a narrower-bandwidth (MHz) and longer coherence time (0.1–1.0 *μs*). Last but not least, the quality of imaging can be well controlled by multiple parameters.

## Basic Theory of Triple-Mode Cone Emission

**Figure 1:**
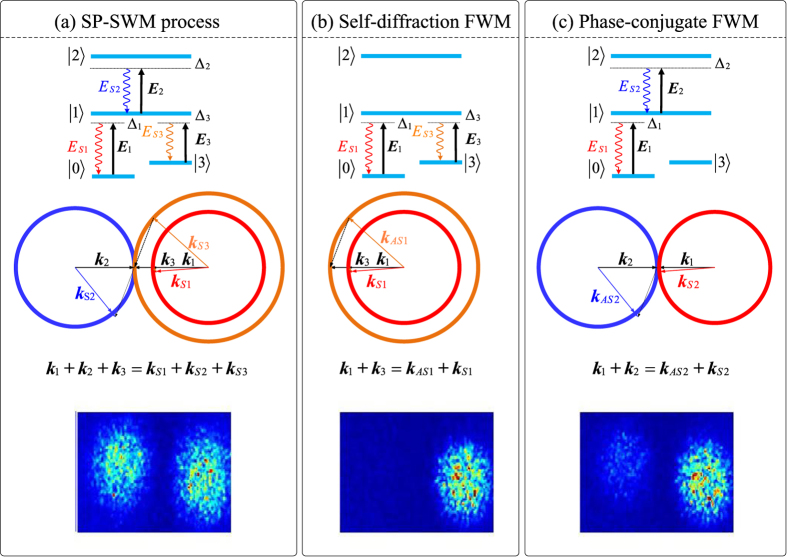
Scheme of reverse Y-type atomic configuration for SP-SWM, including phase matching ring and spots corresponding to such triple-mode cone emission. (**b**,**c**) lambda-type and ladder-type atomic configuration for self-diffraction and phase-conjugate SP-FWM, respectively, including phase matching ring and spots corresponding to those two-mode cone emission.

A theoretical scheme for the preparation triple-mode by SP-SWM is carried out in ^85^Rb atomic ensemble. As shown in Fig. 1(a), the energy levels of 5S_1/2_(F = 3), 5P_3/2_(F = 3), 5D_5/2_, and 5S_1/2_(F = 2) forming the reverse Y-type four-level atomic system^29^ are corresponding to 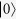, 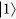, 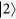, and 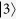, respectively. In this energy level system, three high intensity pumping fields 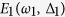, 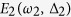, and 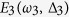 are used to coupling with 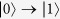, 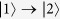 and 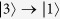, respectively. Therefore, non-degenerate SP-SWM process is excited and three quantum correlated signals *E*_*S*1_, *E*_*S*2_, and *E*_*S*3_ are generated, satisfies the phase-matching condition k_1_ + k_2_ + k_3_ = k_s1_ + k_s2_ + k_s3_, as the phase-matching ring configuration shown in Fig. 1(a).

In this reverse Y-type system, via the perturbation chain 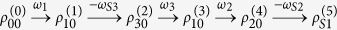, the density matrix element 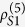 for the generated signal *E*_*S*1_ can be obtained as


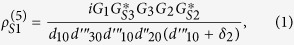


where 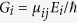 is the Rabi frequency, here *μ*_*ij*_ is electric dipole moment between energy state 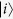 and 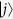, and 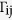 is the transverse decay rate, d_10_ = Γ_10_ + iΔ_1_, 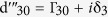, 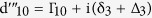, and 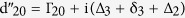. Similarly, we can obtain 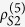 and 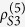 for *E*_*S*2_ and *E*_*S*3_ via the perturbation chains 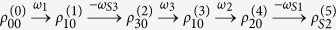 and 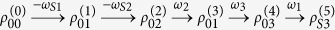 respectively, and the corresponding expressions for *E*_*S*2_ and *E*_*S*3_ are


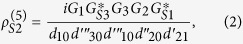



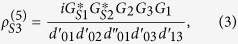


where 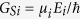 is Rabi frequency of *E*_*Si*_(*i* = 1, 2, 3), *δ*_*i*_ is small fluctuation around *ω*_*j*_ (i = 1, 2, 3 j = S1, S2, S3), with 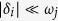. 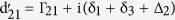, 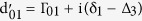, 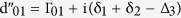, 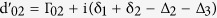, 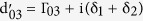, and 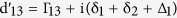. Furthermore, the nonlinear gain is proportional to the corresponding density matrix elements 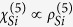.

**Figure 2:**
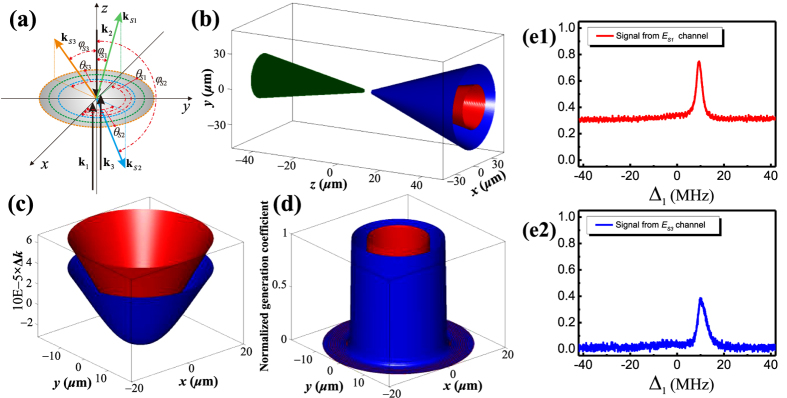
(**a**) Spatial phase matching for SP-SWM processes. (**b**) Cone emission schematic diagram according to Eqs (1, 2, 3, 4, 5). The inner and outer of cones on right-hand side, and the cone on the left-hand side represent k_*S*1_, k_*S*2_ and k_*S*3_, respectively. (**c**) Phase mismatching Δ*k*_*S*1,*S*3_ (x, y, z = 20 m). (**d**) Normalized generation efficient corresponding to (**c**). (**e1**) and (**e2**) are measured intensity of *E*_*S*1_ and *E*_*S*3_ versus Δ_1_ in the SWM process.

The fluctuations of the triple-modes *E*_*S*1_, *E*_*S*2_, and *E*_*S*3_ generated here have zero on average and quantum correlated with each other, That is to say, if there is no seeding to this SP-SWM process, the output states are the triple-mode squeezed vacuum states. By adjusting one of the SP-SWMs, the other two will be affected. For the SP-SWM, as shown in Fig. 2(a), any one of the three polar angles (*φ*_*S*1_, *φ*_*S*2_ or *φ*_*S*3_) can be represented by the other two, and we cannot get the dependent phase mismatching conditions for *E*_*S*1_, *E*_*S*2_ or *E*_*S*3_, which is much more complex than the self-diffraction and phase-conjugate SP-FWM cases. In order to investigate the quantum-correlated of three-photon cone emissions, we fix the polar angle of *k*_*s*2_ to be 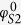, which makes the other two polar angles (*φ*_*s*1_, and *φ*_*s*3_) to be fixed relatively. According to the configuration shown in Fig. 2(a), we can get the phase mismatching conditions for *E*_*S*1_ and *E*_*S*3_ as following,


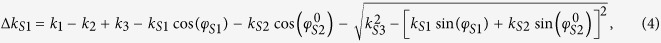



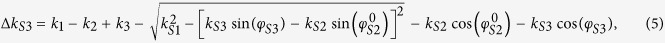


As we know that the fifth-order nonlinear susceptibility highest if the incident light is set collinear 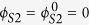, so the nonlinear conversion efficiency is the highest as collinear. However, the generated SP-SWM is buried in strong fluorescence background due to the resonance fluorescence signal is also strongest in this direction. To ensure high conversion efficiency of SP-SWM and suppressed collinear resonance fluorescence, so we set 
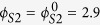
. By setting 
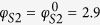
, now we can investigate SP-SWM three-mode cone emission based on Eqs (1, 2, 3, 4, 5), and the main results are shown in Fig. 2(b). According to the requirements spatial phase matching conditions discussed in Fig. 2(a), *E*_*S*3_ (the outer cones) and *E*_*S*1_ (inner cones) propagate along the same direction (the right-hand side ones), and the cones of *E*_*S*2_ propagate along the opposite directions respect to *E*_*S*1_ and *E*_*S*3_ (the left-hand side one).

In light that *E*_*S*2_ is set at the place with the highest emission efficiency intentionally (Δ*k*_*s*2_ = 0 at 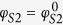), we only display the phase mismatching conditions and the normalized generation efficient of k_*s*1_ and k_*s*3_, which are in Fig. 2(c,d), respectively. According to Eqs (4 and 5), we display the phase mismatching Δ*k*_*s*1,*s*3_ versus transverse coordinates x and y at z = 10 m in Fig. 2(c). It is clear to see that x and y located in a circle is corresponding to Δ*k*_*s*1,*s*3_ = 0, where the six beams are completely phase matching, leading to the largest efficiency to generate the SP-FWMs. However, as x and y deviate the circle, the generation coefficient of the SP-FWMs will reduce greatly for Δ*k*_*s*1,*s*3_ ≠ 0. Therefore, the intensities of *E*_*S*1_ and *E*_*S*3_ are the largest on the circle, and decrease sharply deviate from the circle. To make this problem clearly, we display the normalized generation efficient in Fig. 2(d). It can be seen that the peaks of the normalized generation efficient are on the circles which coincide with the circles Δ*k*_*s*1,*s*3_ in Fig. 2(c). When the propagation distance change, the radii of the circles are also increased monotonously, so we can obtain the circles enlarged along with the increment of propagation distance as shown in Fig. 2(b). The measured intensity of *E*_*S*3_ and *E*_*S*1_ versus Δ_1_ in such SP-SWM process is shown in Fig. 2(e1,e2), where Fig. 2(e1) is measured intensity of *E*_*S*1_ (^85^*Rb*, *F* = 3→*F′* transition) and Fig. 2(e1) is measured intensity of *E*_*S*2_ (^85^*Rb*, *F* = 2→*F′* transition).

On the other hand, a self-diffraction FWM process will be generated in the lambda-type sub-system 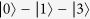 if only *E*_1_ and *E*_3_ are used to driving 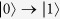 and 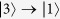, respectively. As shown Fig. 1(b), the cone emissions of *E*_*S*1_ and *E*_*AS*1_ propagate along the same direction through the medium as the requirement of the phase-matching conditions k_1_ + k_3_ = k_*S*1_ + k_*AS*1_. As *E*_*S*1_ and *E*_*AS*1_ in Fig. 1(b) behave like triple-mode SP-SWM if the generated *E*_*S*2_ is removed, therefore, the phase mismatching Δ*k*_*S*1,*AS*1_ and normalized generation efficient also exhibit similar behaviors. In this lambda-type subsystem, via the pathway 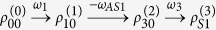 and considering the dressing effect of *E*_3_, the density matrix element for *E*_*S*1_ is


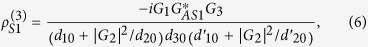


where d_20_ = Γ_20_ + i(Δ_1_ + Δ_2_), d_30_ = Γ_30_ + iδ, 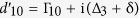, 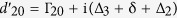, and *δ* is small fluctuation around *ω*_*i*_, with 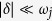
(i = S_1_, AS_1_, S_2_, AS_2_). Similar to 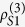, we can obtain the density matrix element for 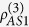 via the pathway 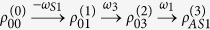 as


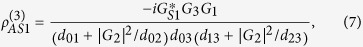


where d_01_ = Γ_01_ − i(Δ_3_ + δ), d_02_ = Γ_02_ − i(Δ_3_ + δ + Δ_2_), d_03_ = Γ_03_ − iδ), d_13_ = Γ_13_ + i(Δ_1_ − δ), and d_23_ = Γ_23_ + i(Δ_1_ + Δ_2_ − δ).

However, if only *E*_1_ and *E*_2_ are applied to driving 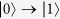 and 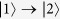 in the ladder-type sub-system 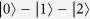 (Fig. 1 (c)), respectively, a phase-conjugate FWM process will be occurred. Different from the self-diffraction FWM cone emission, the phase conjugate FWM cone emissions propagate along the opposite directions for the phase-matching conditions k_1_ + k_2_ = k_*S*2_ + k_*AS*2_ (Fig. 1 (c)). And the radii of the circles increase along the increment of the propagation distance, which make the k_*S*2_ and k_*AS*2_ cones form along the positive and negative propagation directions, respectively. In this ladder-type sub-system 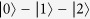, via the pathway 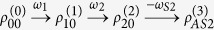 and considering the dressing effect of *E*_3_, the density matrix element for *E*_*AS*2_ can be written as


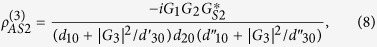


where 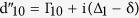, 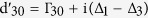, and 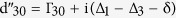. Similarly, the density matrix element for *E*_*S*3_ can be also obtained via the pathway 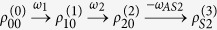:


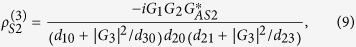


where d_21_ = Γ_21_ + i(δ + Δ_2_) and d_23_ = Γ_23_ + i(Δ_2_ ± Δ_3_ + δ). The measured spots corresponding to SP-SWM, phase-conjugate FWM, self-diffraction FWM are shown in the bottom of Fig. 2(a–c), respectively. When there is no seeding to those SP-FWM process (both self-diffraction FWM and phase-conjugate FWM), the output states are the two-mode squeezed vacuum states and the generated signals are quantum correlated.

Before going to next section, let us pay attention on the comparison between our SP-SWM models and standard FWM scheme. In our theoretical model, SP-SWM process is used to generate quantum correlated triple-mode beams, such process can be considered as the cascading the phase-conjugate FWM and self-diffraction FWM. To be more specific, when pumping fields *E*_1_ with intensity *I*_1_ is seeded into atomic ensemble and coupled with 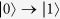, for a gain G created by *E*_2_, twin beams *E*_*S*1_ and *E*_*AS*1_ are simultaneously generated via the self-diffraction FWM process. The intensities of these twin beams *E*_*S*1_ and *E*_*AS*1_ are *I*_*S*1_ = *GI*_1_ and *I*_*AS*1_ = (*G* − 1)*I*_1_, respectively. Although the total power of the twin beams *E*_*S*1_ and *E*_*AS*1_ are significantly amplified, the variance of the relative intensity difference *I*_*S*1_ − *I*_*AS*1_ between them remains unchanged after the amplification. As a result, the relative intensity difference of beams *E*_*S*1_ and *E*_*AS*1_ is squeezed compared with the corresponding shot noise limit (SNL) by an amount of 1/(2*G* − 1). Then, one of the twin beams (say *E*_*S*1_ as shown in Fig. 1(b)) is involved in a phase-conjugate FWM, where the output beam *E*_*S*2_ is amplified and a conjugate beam (*E*_*AS*1_) is simultaneously generated for gain *G* created by *E*_3_. The intensities of these two newly generated twin beams (*E*_*S*2_ and *E*_*AS*1_) are *I*_*S*2_ = *G*^2^*I*_1_ and *I*_*AS*2_ = *G*(*G* − 1)*I*_1_, respectively. If one calculates the intensity-difference noise of the three generated beams (*E*_*AS*1_, *E*_*S*1_ and *E*_*AS*2_), given by *I*_*S*2_ − *I*_*AS*2_ − *I*_*S*1_ and compares it with the corresponding SNL, one will find that the degree of intensity-difference squeezing of the triple beams is given by 1/(2*G*^2^ − 1), and the amount of squeezing in our triple-mode case is significantly increased compared with two-mode squeezing. As a matter of fact, the SP-SWM is generated from internal cascading the self-diffraction and phase-conjugate FWM. Compared with the single self-diffraction or phase-conjugate FWM, the amount of squeezing from SP-SWM is increased from 1/(2*G* − 1) to 1/(2*G*^2^ − 1) due to such cascading effect. In other words, by increasing the number of quantum modes, the quantum correlation is also enhanced in our system. Another advantage of our system is the phase insensitivity that makes it possible to easily extend our system to a large number of modes, as it does not require relative phase stability between all the parametric amplification processes.

## Multi-Mode Squeezing in Ring Cavity

**Figure 3:**
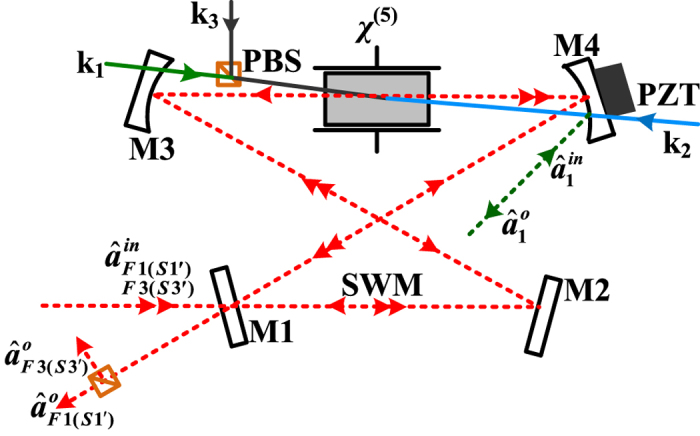
The implementation of multi-mode squeezing via SP-MWM.

Now we theoretically study optical squeezing via multi-wave mixing (MWM) process in an atomic ensemble-cavity coupled system. As shown in Fig. 3, here the ring cavity is formed by four mirrors with a longitudinal cavity length 17 cm. The mirrors M3 and M1 are input and output mirrors with a radius of 50 mm, and the reflectance r3 (r1) and transmittance t3 (t1) coefficient fulfill the constraint condition (i = 1, 3), while M2 and M4 are highly reflection mirrors. Cavity mode scanning and locking can be implemented by a piezoelectric transducer (PZT) behind M4. The length of the atomic vapor cell with the Brewster windows is La = 7 cm, where the atomic vapor cell is wrapped in *μ*-metal sheets to shield from external magnetic fields, and a heat tape is placed outside the sheets for controlling the temperature. Since we do not consider Doppler effects in this paper, our analysis is also suitable for standing-wave cavity. For the limit of cavity, the conical emission is disappeared. However, if paired photons or three photons are prepared simultaneously, the squeezing between them still exists. Now, we apply the basic theory to study optical squeezing via MWM process.

**Figure 4:**
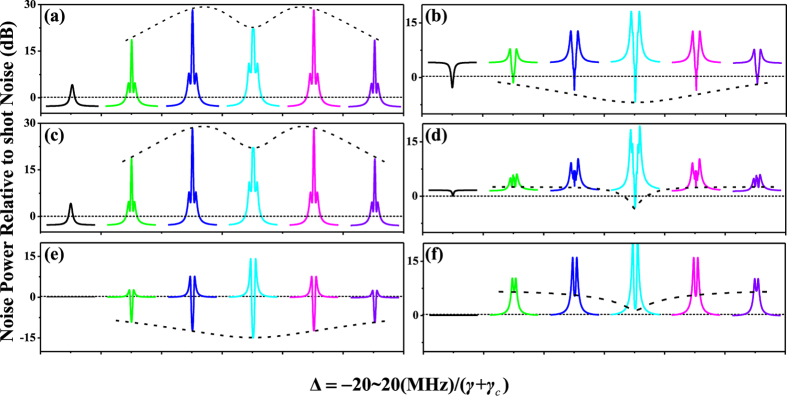
The theoretically calculated squeezing of three-mode versus Δ/(*γ* + *γ*_*c*_) with different Δ_1_/(*γ* + *γ*_*c*_). (**a**–**f**) display the quantum noise variances of the amplitude quadrature summation (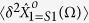,(**a**) 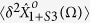 (**c**), 
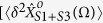
 (**e**)) and phase quadrature summation (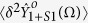 (**b**) 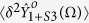 (**d**),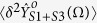 (**f**)). The first curve in each case is the result without any dressing fields, and the following five curves from left to right are noise power spectra with Δ_1_/(*γ* + *γ*_*c*_) = −20, −10, 0, 10, and 20, respectively. The dashed curves are the profiles of noise variances versus Δ_1_/(*γ* + *γ*_*c*_) with Δ/(*γ* + *γ*_*c*_) = 0. The dotted lines are the SNL of the corresponding quadrature.

The quadrature amplitude summations components and quadrature phase summations components are plotted in Fig. 4 by scanning Δ/(*γ* + *γ*_*c*_) at different Δ_1_/(*γ* + *γ*_*c*_), where Fig. 4(a,c,e) are quadrature amplitude summations 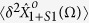, 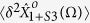, and 
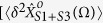
, and Fig. 4(b,d,f) are the quadrature phase summation 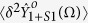, 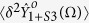, and 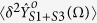, respectively. In Fig. 4, the dotted-line is obtained by blocking the pump fields so as the fifth-order nonlinear process is not active, and the first solid curve in each panel is obtained by injected squeezed vacuum states to cavity and the atomic coupling system, all those (dotted-line and the first solid curve in each panel) can be used as a baseline to examine the effect of the nonlinear process inside cavity on the output states. Compared with the squeezed vacuum states, we can see from Fig. 4(b,e) that the variance of quadrature amplitude summation 
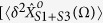
 and quadrature phase summation 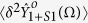 are far below the shot-noise limit (SNL) as well as the first solid curve in each panel in a wide frequency range of Δ_1_/(*γ* + *γ*_*c*_). Therefore, it is enough to assert that 
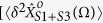
 and 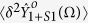 are further squeezed for the influence of fifth-order nonlinear gain. However, the variance of 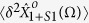 and 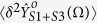 becomes noisier than the input squeezed states, as shown in Fig. 4(a,f), for almost all curve are above the SNL. This phenomenon is in line with Heisenberg uncertainty relationship, if the quantum noise variance of quadrature amplitude (Fig. 4(a)) becomes noisier, the variance of quadrature phase (Fig. 4(b)) will be squeezed, and vice versa as shown in Fig. 4(e,f).

On the other hand, we can see from of Fig. 4(a,c) that the evolutions of the quadrature amplitude noise variances 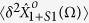 and 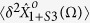 show an AT-splitting (the dashed curve). and the noisy degree are amplified, which is different from Fig. 4(e). The main difference can be explained by the interaction Hamiltonian 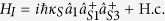 describing the SP-SWM process, where the generation of any two photons 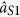 and 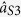 were accompanied by the annihilation a probe photon 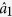. Therefore, in this nonlinear SP-SWM process, 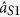 and 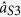 have similar quantum characteristics, which is different from 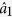. In other words, 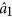 and 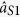 (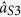) in competition with each other. Contrary to this, for 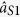 and 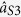 have similar quantum characteristics, the noisy degree is significantly squeezed for anti-amplification of nonlinear gain. In addition, with the sequential (or nested)-cascade type of double dressing as well as 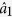 and 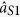 (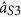) have different quantum characteristics, the tri-peak AT structure of each curve in Fig. 4(a,c) are observed, and the noisy degree of 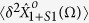 and 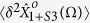 are enhanced for amplification of nonlinear gain. These results indicate that the squeezing degree or the noisy degree can be effectively modulated by the nonlinear gain *κ*_*S*_ as well as the quantum characteristics of the triple-mode.

We now consider the self-diffraction SP-FWM (Fig. 1 (b)) process in the ring cavity shown in Fig. 3, where the generated SP-FWM signals *E*_*S*1_ and *E*_*AS*1_ are propagate along the cavity axis with the same direction forming two cavity modes, and detected by one APD device. As the polarizations of two modes are perpendicular, one can record them independently by putting a polarizer before the APD device and rotating it. On the other hand, in the phase-conjugate SP-FWM process, these two SP-FWM signals will form two modes with different directions, which can be detected by two APD devices. Although the phase-conjugate and self-diffraction SP-FWM are described by different nonlinear coefficient *κ*_1_ and *κ*_2_, respectively, these SP-FWM signals (both the phase-conjugate and self-diffraction) have the similar quantum characteristics except propagation along different directions. Therefore, the motion equations of such two SP-FWM signals can be written with unified form.


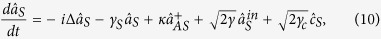



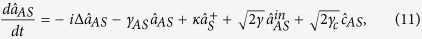


where 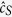 and 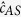 are the coupled vacuum modes.

**Figure 5:**
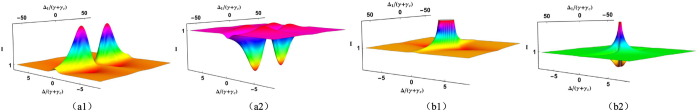
Theoretically calculated two-mode squeezing based on Eqs (10 and 11) versus Δ/(*γ* + *γ*_*c*_) and Δ_1_/(*γ* + *γ*_*c*_), with coherent fields injected, (**a1**,**a2**) are 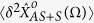 and 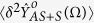 with the dressing effect of *E*_2_, respectively. (**b1**,**b2**) are 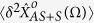 and 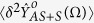 without *E*_2_, respectively.

Considering the Fourier transform of Eqs (10 and 11) and boundary condition 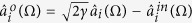, the quantum noise variance of quadrature amplitude summation 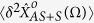 and quadrature phase summation 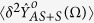 at output can be investigated by scanning Δ/(*γ* + *γ*_*c*_) and Δ_1_/(*γ* + *γ*_*c*_) simultaneously. To invest noise fluctuation of cavity mode under various injection fields, here EPR field and coherent field is injected into the ring cavity to make a comparison. Firstly, we adopt coherent fields as the injected fields. The quantum noise variances of 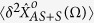 and 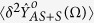 are illustrated in Fig. 5(a1,a2), respectively. From Fig. 5(a1,a2), we can see that only 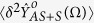 is lower than SNL as Δ_1_/(*γ* + *γ*_*c*_) scanned from negative to positive, where the maximum squeezing is corresponding to the location of reverse AT splitting created by *E*_2_, that is, the position of the dark state. While, the quantum noise variances of 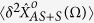 become noisier as Δ_1_/(*γ* + *γ*_*c*_) is scanned in the vicinity of the dressed state. On the other hand, except without considering the dressing effect from *E*_2_, other conditions in Fig. 5(b1,b2) are same as those in Fig. 5(a1,a2), respectively. In comparison with the cases with and without dressing effect of *E*_2_, the AT splitting in quadrature amplitude and the reverse AT splitting in quadrature phase are disappears, and the location of maximum noisier and squeezing are moved to Δ_1_/(*γ* + *γ*_*c*_). We can see that the dressed state can effectively control the squeezing process. Therefore, the influence of the AT splitting of *E*_2_ on the squeezing is obvious.

**Figure 6:**
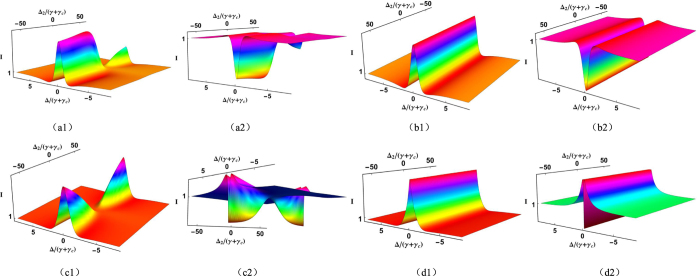
The theoretically calculated squeezing of two-mode versus Δ/(*γ* + *γ*_*c*_) and Δ_2_/(*γ* + *γ*_*c*_) synchronously. By setting Δ_1_/(*γ* + *γ*_*c*_) = 15, (**a1**,**a2**), (**b1**,**b2**) are the amplitude quadrature summation and phase quadrature summation with (without) considering the dressing effect of *E*_2_. By setting Δ_1_/(*γ* + *γ*_*c*_) = 0, (**c1**,**c2**) (**d1**,**d2**) are the amplitude quadrature summation and phase quadrature summation with (without) considering the dressing effect of *E*_2_.

Then, by setting Δ_1_/(*γ* + *γ*_*c*_) = 15 (Fig. 6(a1–a2,b1–b2)) and Δ_1_/(*γ* + *γ*_*c*_) = 0 (Fig. 6(c1–c2,d1–d2)), respectively, and scanning Δ/(*γ* + *γ*_*c*_) as well as Δ_2_/(*γ* + *γ*_*c*_) synchronously, the suppression and enhancement role of dressed state on the quantum noise variance of 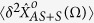 and 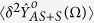 are studied. In the case of Δ_1_/(*γ* + *γ*_*c*_) = 15, as shown in Fig. 6(a1), the quantum noise variance of 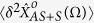 becomes noisier and no squeezing when Δ_1_/(*γ* + *γ*_*c*_) is scanned from positive to negative. Specifically speaking, the noise fluctuation of 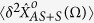 is significantly enhanced in the region Δ_2_ < 0, and suppressed in the region Δ_2_ > 0, which is corresponding enhancement (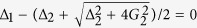) and suppression (Δ_1_ = Δ_2_ = 15) conditions of the dressed state, respectively. It should be emphasized that 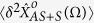 is still no squeezing in suppression region Δ_2_ > 0 for the curve is above the SNL. However, the situation is exactly the opposite in terms of the quadrature phase summation, as shown in Fig. 6(a2), where the profiles of quantum noise variances show that 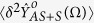 is squeezed when Δ_2_/(*γ* + *γ*_*c*_) is scanned, and the degree of squeezing get its maximum in the region Δ_2_ < 0. Fig. 6(b1,b2) are same as those in Fig. 6(a1,a2) except without considering the dressing effect from *E*_2_. In comparison the cases with and without *E*_2_, the quantum noise variances of the 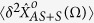 and 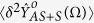 is unchanged as Δ_2_/(*γ* + *γ*_*c*_) is scanned. For the case Δ/(*γ* + *γ*_*c*_) = 0, we take the same method to study the influence of dressed state on two-mode squeezing. As shown in Fig. 6(c1,c2), the quantum noise variances of the 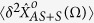 and 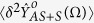 at Δ = 0 exhibit a pure suppression and a pure enhancement, respectively. It is worth mentioning that the suppression conditions are all Δ_1_ = Δ_2_. If the dressing effect of *E*_2_ can be neglected, the quantum noise variances are also not affected as shown in Fig. 6(d1,d2). It is clear see that two-mode squeezing can be effectively controlled by the suppression and enhancement of dressed state.

**Figure 7:**
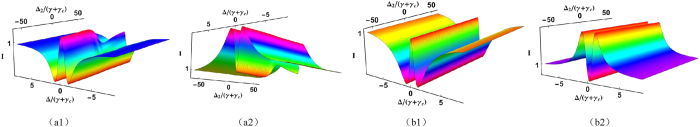
Theoretically calculated two-mode squeezing versus Δ/(*γ* + *γ*_*c*_) and Δ_2_/(*γ* + *γ*_*c*_), with EPR fields injected, (**a1**,**a2**) are 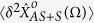 and 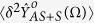 with dressing effect of *E*_2_, respectively. (**b1**,**b2**) are 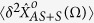 and 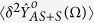 without *E*_2_, respectively.

Finally, the influence of dressed state on the squeezing with Einstein-Podolsky-Rosen (EPR) fields injected is shown in Fig. 7(a1–a2), where Δ/(*γ* + *γ*_*c*_) and Δ_2_/(*γ* + *γ*_*c*_) are scanned with Δ_1_/(*γ* + *γ*_*c*_) = 15. As shown in Fig. 7(a1), the variance of 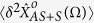 becomes noisier when Δ_2_/(*γ* + *γ*_*c*_) is scanned from positive to negative, and the profile of the variances (showing enhancement in Δ_2_ < 0 and suppression in Δ_2_ > 0) is same as the situation when coherent fields injected is considered. In addition, although 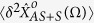 is injected with squeezed fields and suppressed at Δ_2_ > 0, there is no squeezing in all region. As mentioned above, for the influence of dressed state as well as injected with squeezed fields, the variance of 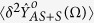 shown in Fig. 7(a2) is significantly squeezed compared with the injecting coherent fields. To be specific, 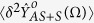 get its maximum squeezing value at 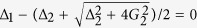, and minimum squeezing value at Δ_1_ = Δ_2_ = 15. On the other hand, the quantum noise variances of the 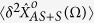 and 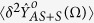 do not change versus Δ_1_/(*γ* + *γ*_*c*_) if the dressing effect from is absent. However, 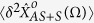 and 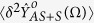 shown in Fig. 7(b1,b2) have a higher squeezing level than those shown in Fig. 6(b1,b2) for injected with squeezed fields. It is worth mentioning that our scheme has following advantages. First, due to the near- and on-resonance nonlinear optical processes can be enhanced by atomic coherence technique, so the generation efficiency of SP-SWM and degree of triple of squeezing (entanglement) can be significantly enhanced by manipulating nonlinear susceptibility and quantum gain. Second, due to electromagnetically induced transparency (EIT) window (MHz), not only the resonance absorption is eliminated, but narrow bandwidth signals at low light level is obtained as well. So compared with correlated photon pairs from SPDC, which have characteristics of very wide bandwidth (THz) and ultra-short coherence time (ps). Our scheme has a narrower-bandwidth (MHz) and longer coherence time (0.1–1.0 *μs*). Last but not least, the quality of imaging can be well controlled by multiple parameters.

## Conclusion

In summary, we have theoretically investigated the multi-mode quantum noise squeezing and amplification with MWM signals in a ring cavity filled with rubidium vapors. It is found that the squeezing and amplification of quantum noise can be effectively modulated by dressed state and nonlinear gain coefficient. Specifically, nonlinear gain leads to the optical squeezing as well as nonlinear AT splitting of cavity mode, and dressed state can be used to reshape the efficiency as well as the location of maximum squeezing point. In optical squeezing, the physical properties of dressed state and nonlinear gain are quite similar. It is worth mentioning that our system has following advantages, first, the amount of squeezing from SP-SWM is significantly improved compared with self-diffraction and phase-conjugate FWM. Second, intensity-difference squeezing from SPDC has the characteristics of very wide bandwidth (THz) and ultra-short coherence time (ps), however, a narrower-bandwidth (MHz) and longer coherence time (0.1–1.0 *μs*) is implemented from SP-SWM. Such as long coherence time allows us to access and manipulates the spatial squeezing directly. Third, intensity-difference squeezing can be well controlled by multiple parameters. One of the main advantages of our system is that can be directly used in multichannel quantum imaging, and the quality of imaging, including the contrast and resolution, is significantly improved compared with the two-mode squeezing state. The proposed method is also used as implementation of triple-mode entangled source to achieve multi-channel communication.

## Theoretical Models

In this part, a new scheme via fifth-order nonlinear channels to produce triple mode squeezed state is proposed as shown in Fig. 3, where *E*_1_, *E*_2_, and *E*_3_ are injected into the ring cavity and coupled to 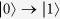, 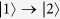, and 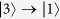, respectively. Considering the assumption that the fields *E*_2_, and *E*_3_ are much larger than the probe *E*_1_, two SWM (*E*_*S*1_ and *E*_*S*3_) photons are generated, which is accompanied by the annihilation a probe photon *a*_1_ and three pumping field photon with phase matching condition satisfy k_*S*1_ = k_1_ + k_2_ − k_2_ + k_3_ − k_*S*3_ and k_*S*3_ = k_1_ + k_3_ − k_*S*1_ + k_2_ − k_2_. In this process, *E*_1_, *E*_*S*1_ and *E*_*S*3_ are treated as quantum fields, while *E*_2_, and *E*_3_ are considered as classical fields, therefore, the interaction Hamiltonian describing this process can be expressed as 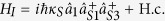, where 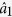, 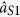 and 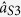 are the annihilation operator of triple cavity modes *E*_1_, *E*_*S*1_ and *E*_*S*3_. The fifth-order nonlinear coefficients *κ*_*S*_ described by the nonlinear gain in SWM processes, is proportional to 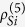. On the other hand, the Hamiltonian for the probe field is 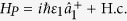, where *ε*_1_ is the amplitude of the probe field. Therefore, taking into account the loss, and nonlinear gain, we can obtain the equations for three cavity modes as following:


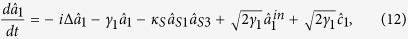



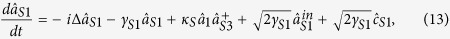



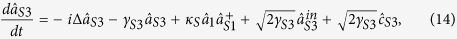


where Δ is the cavity detuning, 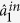, 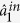 and 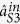 denote the injected fields at *E*_1_, *E*_*S*1_ and *E*_*S*3_ channels, respectively. 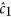, 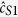, and 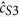 are the vacuum modes coupled with the corresponding cavity modes 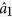, 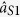 and 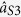, and *γ*_1_, *γ*_*S*1_, and *γ*_*S*3_ denote the dimensionless damping rate which are related to the amplitude reflection and transmission coefficients of the input and output couplers of the optical cavity.

Without loss of generality, one can decompose the system variables (including the resonant cavity modes, the injected fields, and vacuum modes) into their steady-state values and small fluctuations around the steady-state values, for instance, 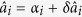, 
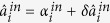
, 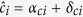 (i = 1, S1, S3), where *α*_*i*_, 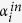 and *α*_*ci*_ indicate the mean values of the corresponding fields, and 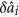, 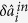, and 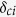 demonstrate small fluctuations around the steady-state values. The injected probe field is a coherent field, and its fluctuations are the same as the vacuum fluctuations, so 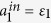. However, the generated cavity modes and coupled vacuum modes have zero mean values, i.e., 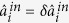 and 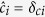.

The steady-state solutions can be obtained by letting *dα*_*i*_/*dt* = 0 (*i* = 1, *S*1, *S*3), and throwing away the vacuum fluctuations as well as the cavity detuning in Eqs (12, 13, 14). Therefore, the steady state value of the triple cavity mode satisfies the following equations


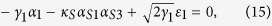



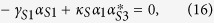



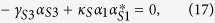


In order to simplify the calculation, we assume that 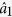, 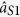 and 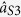 are nearly frequency degenerate, leading damping rates to be identical (*γ*_1_ = *γ*_*S*1_ = *γ*_*S*3_), Now, the steady-state values of of triple cavity modes have following value:


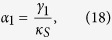



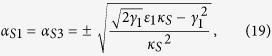


By means of linearized analysis procedure, small fluctuations around of triple cavity mode can be obtained,


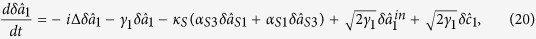



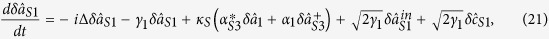



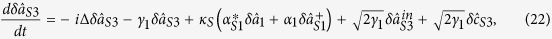


Now, we need the fluctuations of quadrature amplitude (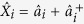) and quadrature phase (
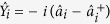
) components to study squeezing characteristics between triple modes. Considering the Fourier transform of the operators, Eqs (20, 21, 22) can be recasted in terms of the quadrature amplitude and phase operators:


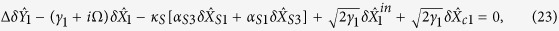



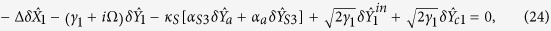



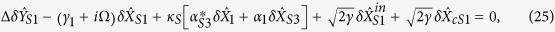



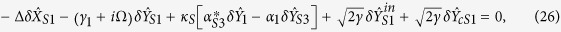



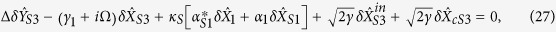



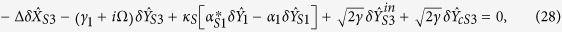


where Ω is the analysis frequency. Now, these equations can be solved in the frequency domain under the boundary condition 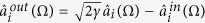, and the spectra of squeezing characteristics between triple modes in terms of the input fluctuation can be obtained analytically. Whats more, the fluctuations of quadrature amplitude summation is same as the quadrature phase difference of any two output modes, and the quadrature amplitude difference is same as the quadrature phase summation of any two output modes, therefore, we will investigate the triple modes squeezing by means of amplitude and quadrature phase summation.

## Additional Information

**How to cite this article**: Wen, F. *et al.* Triple-mode squeezing with dressed six-wave mixing. *Sci. Rep.* **6**, 25554; doi: 10.1038/srep25554 (2016).

## References

[b1] Akamatsu D., Akiba K. & Kozuma M. Electromagnetically Induced Transparency with Squeezed Vacuum. Phys. Rev. Lett. 92, 203602 (2004). 15169353 10.1103/PhysRevLett.92.203602

[b2] Braunstein S. L. & van Loock P. Quantum information with continuous variables. Rev. Mod. Phys. 77, 513–577 (2005).

[b3] Bowen W. P. *et al.* Experimental investigation of continuous-variable quantum teleportation. Phys. Rev. A 67, 032302 (2003).

[b4] Vahlbruch H. *et al.* Observation of Squeezed Light with 10-dB Quantum-Noise Reduction. Phys. Rev. Lett. 100, 033602 (2008). 18232978 10.1103/PhysRevLett.100.033602

[b5] Aoki T. *et al.* Experimental Creation of a Fully Inseparable Tripartite Continuous-Variable State. Phys. Rev. Lett. 91, 080404 (2003). 14525227 10.1103/PhysRevLett.91.080404

[b6] Menicucci N. C., Flammia S. T. & Pfister O. One-Way Quantum Computing in the Optical Frequency Comb. Phys. Rev. Lett. 101, 130501 (2008). 18851426 10.1103/PhysRevLett.101.130501

[b7] Coelho A. S. *et al.* Three-Color Entanglement. Science 326, 823–826 (2009). 19762598 10.1126/science.1178683

[b8] Sokolov I. V. & Kolobov M. I. Squeezed-light source for superresolving microscopy. Opt. Lett. 29, 703–705 (2004). 15072364 10.1364/ol.29.000703

[b9] Burnham D. C. & Weinberg D. L. Observation of Simultaneity in Parametric Production of Optical Photon Pairs. Phys. Rev. Lett. 25, 84–87 (1970).

[b10] Kwiat P. G. *et al.* New High-Intensity Source of Polarization-Entangled Photon Pairs. Phys. Rev. Lett. 75, 4337–4341 (1995). 10059884 10.1103/PhysRevLett.75.4337

[b11] Slusher R., Hollberg L., Yurke B., Mertz J. & Valley J. Observation of squeezed states generated by four-wave mixing in an optical cavity. Phys. Rev. Lett. 55, 2409 (1985). 10032137 10.1103/PhysRevLett.55.2409

[b12] Qin Z. *et al.* Experimental generation of multiple quantum correlated beams from hot Rubidium vapor. Phys. Rev. Lett. 113, 023602 (2014). 25062179 10.1103/PhysRevLett.113.023602

[b13] Qin Z., Cao L. & Jing J. Experimental characterization of quantum correlated triple beams generated by cascaded four-wave mixing processes. Appl. Phys. Lett. 106, 211104 (2015).

[b14] Fleischhauer M., Imamoglu A. & Marangos J. P. Electromagnetically induced transparency: Optics in coherent media. Rev. Mod. Phys. 77, 633–673 (2005).

[b15] Akamatsu D. *et al.* Ultraslow Propagation of Squeezed Vacuum Pulses with Electromagnetically Induced Transparency. Phys. Rev. Lett. 99, 153602 (2007). 17995164 10.1103/PhysRevLett.99.153602

[b16] Hètet G. *et al.* Delay of squeezing and entanglementusing electromagnetically inducedtransparency in a vapour cell. Opt. Express 16, 7369–7381 (2008). 18545442 10.1364/oe.16.007369

[b17] Arikawa M. *et al.* Observation of electromagnetically induced transparency for a squeezedvacuum with the time domain method. Opt. Express 15, 11849–11854 (2007). 19547547 10.1364/oe.15.011849

[b18] Honda K. *et al.* Storage and Retrieval of a Squeezed Vacuum. Phys. Rev. Lett. 100, 093601 (2008). 18352709 10.1103/PhysRevLett.100.093601

[b19] Appel J., Figueroa E., Korystov D., Lobino M. & Lvovsky A. I. Quantum Memory for Squeezed Light. Phys. Rev. Lett. 100, 093602 (2008). 18352710 10.1103/PhysRevLett.100.093602

[b20] Simon C. *et al.* Quantum Repeaters with Photon Pair Sources and Multimode Memories. Phys. Rev. Lett. 98, 190503 (2007). 17677612 10.1103/PhysRevLett.98.190503

[b21] Duan L.-M., Lukin M., Cirac J. I. & Zoller P. Long-distance quantum communication with atomic ensembles and linear optics. Nature 414, 413–418 (2001). 11719796 10.1038/35106500

[b22] Grangier P., Slusher R., Yurke B. & LaPorta A. Squeezed-light–enhanced polarization interferometer. Phys. Rev. Lett. 59, 2153 (1987). 10035438 10.1103/PhysRevLett.59.2153

[b23] Shapiro J. H. Optical waveguide tap with infinitesimal insertion loss. Opt. Lett. 5, 351–353 (1980). 19693225 10.1364/ol.5.000351

[b24] Zhang Y., Anderson B. & Xiao M. Efficient energy transfer between four-wave-mixing and six-wave-mixing processes via atomic coherence. Phys. Rev. A 77, 061801 (2008).

[b25] Zhang Y. Q. *et al.* Photonic Floquet topological insulators in atomic ensembles. Laser Photon. Rev. 9, 331–338 (2015).

[b26] Zhang Y. Q., Liu X., Belić M. R., Wu Z. K. & Zhang Y. P. Modulation of the photonic band structure topology of a honeycomb lattice in an atomic vapor. Ann. Phys. 363, 114–121 (2015).

[b27] Ma H. & de Araujo C. B. Interference between third-and fifth-order polarizations in semiconductor doped glasses. Phys. Rev. Lett. 71, 3649 (1993). 10055037 10.1103/PhysRevLett.71.3649

[b28] Wen F. *et al.* Multidressed suppression and enhancement of spontaneous parametric four-wave-mixing processes. J. Opt. Soc. Am. B 31, 2384–2389 (2014).

[b29] Zhang Y. Q. *et al.* Optical cavity squeezing of multiwave mixing via dark states. J. Opt. Soc. Am. B 31, 2792–2801 (2014).

